# Sex-specific associations between estimated glucose disposal rate and cognitive decline in middle-aged and older adults in China: a longitudinal cohort study

**DOI:** 10.3389/fnagi.2025.1544352

**Published:** 2025-02-05

**Authors:** Chun Luo, Shuang Han, Xiaoying Shen, Hao Wu, Jianqing Zhou, Bingyang Liu

**Affiliations:** ^1^Ningbo Medical Center Lihuili Hospital, Ningbo University, Ningbo, China; ^2^Affiliated Hangzhou First People’s Hospital, School of Medicine, Westlake University, Hangzhou, China

**Keywords:** estimated glucose disposal rate, cognitive decline, insulin resistance, sex differences, longitudinal analysis

## Abstract

**Background:**

Insulin resistance (IR) is recognized as a potential modifiable risk factor for cognitive decline, but findings within Asian populations have been inconsistent. Given the high prevalence of dementia and its substantial economic burden in China, large-scale longitudinal studies are essential to elucidate the complex relationship between IR and cognitive function.

**Methods:**

This longitudinal cohort study included 8,734 middle-aged and older adults (median age: 58 years; 53.6% females) from the China Health and Retirement Longitudinal Study (CHARLS), followed from 2011 to 2018. Estimated glucose disposal rate (eGDR) was used to assess IR and was calculated using waist circumference, hypertension status, and HbA1c levels. Participants were categorized into tertiles based on eGDR levels (Tertile 1: lowest; Tertile 3: highest). Cognitive function was calculated as the sum of episodic memory and executive function scores, which was then standardized to a *Z*-score. Linear mixed-effects models and dose-response analyses were performed to evaluate the association between baseline eGDR and cognitive changes in the total population and stratified by sex.

**Results:**

Higher eGDR levels were significantly associated with slower global cognitive decline (Tertile 3 vs. Tertile 1: β = 0.007; 95% CI: 0.000–0.014; *P* = 0.047). This association was stronger in females (Tertile 3 vs. Tertile 1: β = 0.011; 95% CI: 0.002–0.021; *P* = 0.021), while no significant association was observed in males. Dose-response analyses indicated a linear positive relationship between baseline eGDR and global cognitive function in the total population and in females, but not in males. Similar patterns were found for episodic memory and executive function, with significant associations predominantly in females.

**Conclusion:**

Higher eGDR was significantly associated with slower cognitive decline, particularly among women. These findings underscore the potential of eGDR as a marker for identifying and mitigating cognitive decline and highlight the importance of sex-specific strategies to address insulin resistance and promote cognitive health.

## 1 Introduction

China, one of the world’s most populous countries, hosts approximately 16.99 million individuals diagnosed with dementia, accounting for 30% of the global dementia population (Wang et al., 2024). The nation is experiencing a rapidly aging population alongside increasing life expectancy, factors anticipated to further elevate the prevalence of dementia. Over the next 3 decades, the economic burden of dementia in China is projected to reach $296.1 billion, surpassing the estimated burdens in the United States ($233.1 billion) and Japan ($175.8 billion), thereby positioning China as the country with the highest economic impact of dementia globally (Chen et al., 2024). In the absence of curative treatments for dementia, identifying modifiable risk factors that could delay or prevent cognitive decline has emerged as a critical public health priority (Reuben et al., 2024).

Insulin resistance (IR) has garnered significant attention for its potential role in cognitive decline (Affuso et al., 2024). Notably, both lifestyle interventions and pharmacological treatments have been shown to effectively improve IR, highlighting its potential as a modifiable target for intervention (Li M. et al., 2022). Mechanistic studies suggest that IR may accelerate cognitive deterioration through pathways involving chronic inflammation, impaired synaptic activity, neurotransmitter dysregulation, and vascular dysfunction (Cannavale et al., 2021; Kellar and Craft, 2020; Suren Garg et al., 2023; Tumminia et al., 2018). Research conducted within Western populations consistently demonstrates significant associations between IR and cognitive decline (Fava et al., 2017; Hooshmand et al., 2019; Neergaard et al., 2017; Sherzai et al., 2018; Smith et al., 2019) However, studies within Asian populations have yielded limited and inconsistent findings (Ji et al., 2024b; Kong et al., 2018; Lee et al., 2019; Ma and Li, 2017; Yu et al., 2023). For instance, a 6-year longitudinal study among community-dwelling older adults in Korea reported that dynamic increases in IR significantly predicted cognitive decline (Kong et al., 2018). In contrast, another study involving middle-aged and older diabetic patients in Korea found no significant association between baseline IR and cognitive decline (Yu et al., 2023). Additionally, research in a Japanese non-diabetic population suggested that IR might be associated with a reduced risk of dementia (Lee et al., 2019). In China, small cross-sectional studies identified a significant association between IR and cognitive decline among individuals with type 1 diabetes (Ji et al., 2024b) and type 2 diabetes (Ma and Li, 2017). However, these studies were limited by their modest sample sizes of approximately 100–200 participants and the absence of longitudinal data (Ji et al., 2024b; Ma and Li, 2017). These discrepancies may reflect variations in population characteristics. Given the distinct genetic, dietary, and environmental factors in China, along with the high prevalence of dementia and its substantial economic burden, it is crucial to conduct large-scale longitudinal studies in this population to better understand the complex relationship between IR and cognitive function and to identify potential risk factors unique to the Chinese context.

Assessing IR in large-scale population studies presents significant methodological challenges. The hyperinsulinemic-euglycemic clamp is considered the gold standard for measuring IR but is often impractical for epidemiological studies due to its high cost and complexity (James et al., 2020). The Homeostatic Model Assessment of Insulin Resistance (HOMA-IR) offers a more feasible alternative; however, it requires fasting insulin measurements, which are frequently unavailable in community-based studies (Tahapary et al., 2022). Recently, the estimated glucose disposal rate (eGDR) has emerged as a reliable surrogate marker for IR (Epstein et al., 2013). eGDR is calculated using HbA1c levels, waist circumference, and hypertension status, and it demonstrates a strong correlation with glucose uptake rates (M-values) measured by the hyperinsulinemic-euglycemic clamp (*r* = 0.63, *P* < 0.001) (Mertens et al., 2024). Due to its reliance on routine clinical measures, eGDR is both simple to implement and highly suitable for large-scale epidemiological studies (Meng et al., 2023; Peng et al., 2023; Zhang Y. et al., 2024). A cross-sectional study involving over 100 participants indicated that eGDR is associated with cognitive decline in individuals with type 1 diabetes (Ji et al., 2024b).

This study utilizes data from the China Health and Retirement Longitudinal Study (CHARLS), a nationally representative cohort of middle-aged and older adults in China. CHARLS encompasses both urban and rural residents and is distinguished by its large sample size, long-term follow-up, and comprehensive multidomain data collection (Zhao et al., 2014). This dataset provides a robust platform to investigate the relationship between IR and cognitive decline, reflecting China’s unique socioeconomic, cultural, and epidemiological landscape (Zhao et al., 2014). Although CHARLS does not include direct measures of IR, eGDR serves as a validated surrogate marker, offering a feasible approach to evaluate the role of IR in this population. Moreover, sex differences may significantly influence the association between IR and cognitive decline. Research indicates that women, particularly postmenopausal women, are at higher risk for IR due to hormonal changes (De Paoli et al., 2021), and the patterns of cognitive decline differ markedly between men and women (Bloomberg et al., 2023; Tu et al., 2024). Therefore, the present study aims to analyze the relationship between baseline eGDR and cognitive decline over a seven-year follow-up period to explore IR as a potential modifiable risk factor for cognitive decline in the Chinese population. Additionally, stratified analyses by sex will be conducted to examine potential moderating effects on this association.

## 2 Materials and methods

### 2.1 Study design and participants

This study utilized data from the CHARLS, a nationally representative prospective cohort focused on individuals aged ≥ 45 years in China (Zhao et al., 2014). CHARLS employed a multi-stage, stratified probability sampling method, with baseline data collection beginning in 2011 and follow-up assessments conducted every 2–3 years. Data were gathered through face-to-face interviews, laboratory tests, and physical measurements during the 2011 (Wave 1), 2013 (Wave 2), 2015 (Wave 3), and 2018 (Wave 4) waves. Due to disruptions from the COVID-19 pandemic, the 2020 wave included partial data collected *via* video interviews, which was excluded for consistency. This study analyzed data from the 2011 to 2018 waves.

CHARLS collected blood samples for glycated hemoglobin (HbA1c) at baseline and recorded data on hypertension, waist circumference, and cognitive function during each follow-up wave. Participants were excluded if they were younger than 45 years of age or had missing baseline data for hypertension, HbA1c, or waist circumference. Individuals with a history of dementia, Parkinson’s disease, brain atrophy, or mood/mental disorders at baseline were also excluded. Additionally, participants with incomplete cognitive assessments at baseline or no follow-up data on cognitive measurements were excluded.

**FIGURE 1:**
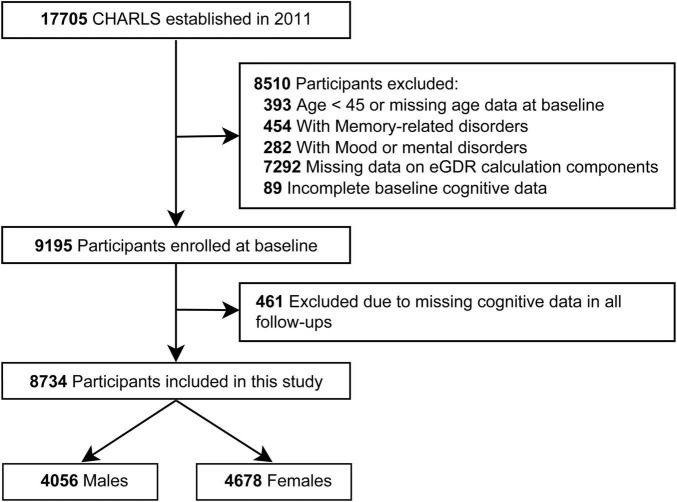
Flow diagram of participant selection.

The final study sample was derived through a rigorous selection process, as outlined in Figure 1. All participants provided informed consent, and the study protocol was approved by the Biomedical Ethics Committee of Peking University (IRB00001052-11015).

### 2.2 Measurement of eGDR

The primary exposure variable in this study was eGDR, calculated using the following formula:


eGDR⁢(mg/kg/min)=21.158-(0.09×WC)-

(3.407×hypertension)-(0.551×HbA1c),

where WC is waist circumference (in cm), hypertension is coded as 1 for “yes” and 0 for “no,” and HbA1c is expressed as a percentage (Zhang Z. et al., 2024). Waist circumference was measured under standardized conditions, with participants wearing light clothing.

Participants were classified as hypertensive if they had a systolic blood pressure (BP) ≥ 140 mmHg, diastolic BP ≥ 90 mmHg, were taking antihypertensive medications, or self-reported a diagnosis of hypertension. Blood samples were collected after overnight fasting, stored at −20°C, and transported to Beijing for HbA1c analysis using standardized procedures.

In this study, lower eGDR scores were assumed to indicate higher insulin resistance.

### 2.3 Measurement of cognitive function

Cognitive function was assessed through face-to-face interviews using standardized tools to evaluate episodic memory and executive function (Lin et al., 2022).

Episodic memory was assessed using immediate and delayed recall tasks. Participants were asked to recall 10 Chinese words immediately after hearing them and again after a 5-min delay. The final episodic memory score was calculated as the average of the immediate and delayed recall scores, with a range of 0–10. Executive function was evaluated through tasks measuring orientation, calculation, and visuospatial ability. Orientation involved identifying the current year, month, date, day of the week, and season, with a score range of 0–5. Calculation was assessed by asking participants to subtract 7 from 100 consecutively 5 times, with a score range of 0–5. Visuospatial ability required participants to redraw a displayed figure accurately, with a score range of 0–1. The total executive function score ranged from 0 to 11.

Overall cognitive function was defined as the sum of the episodic memory and executive function scores, yielding a possible range of 0–21. All raw scores were standardized into *Z*-scores based on baseline means and standard deviations (*SDs*), with higher *Z*-scores indicating better cognitive performance.

### 2.4 Covariates

Potential confounders previously identified as risk factors for cognitive impairment in the Chinese population were included in the analysis (Jia et al., 2020). These factors encompassed demographics, socioeconomic factors, health behaviors, and chronic diseases. Demographic covariates included age and sex (male or female). Socioeconomic factors included area of residence (urban or rural), marital status (married/cohabiting or other), and educational level (elementary school or below, secondary school, or college or above). Health behavior variables included smoking status (non-smokers or smokers), alcohol consumption (non-drinkers or drinkers), and body mass index (BMI). Chronic diseases (yes or no) included dyslipidemia, diabetes, heart disease, and stroke. BMI was calculated as weight (kg) divided by the square of height (m^2^). The diagnoses of dyslipidemia and diabetes were consistent with previous studies (Luo et al., 2023). Dyslipidemia was defined by: (1) total cholesterol ≥ 240 mg/dL, low-density lipoprotein (LDL) ≥ 160 mg/dL, triglycerides ≥ 200 mg/dL, high-density lipoprotein (HDL) < 40 mg/dL, or (2) current use of lipid-lowering medications, or (3) self-reported physician-diagnosed hyperlipidemia. Diabetes was diagnosed based on: (1) fasting plasma glucose ≥ 126 mg/dL, random plasma glucose ≥ 200 mg/dL, or hemoglobin A1c ≥ 6.5%, or (2) current use of hypoglycemic treatment, or (3) a previous diagnosis of diabetes. The presence of heart disease and stroke was determined based on prior diagnosis or current use of relevant medications. Additionally, C-reactive protein (CRP) and hemoglobin levels were included as covariates in the analysis, reflecting inflammatory status and anemia (e.g., iron-deficiency anemia).

### 2.5 Statistical analysis

The study population was divided into three groups based on baseline eGDR tertiles. Analyses were conducted for the total population and separately for males and females. Continuous variables were presented as medians with interquartile ranges (IQRs), and differences between groups were evaluated using the Kruskal-Wallis test. Categorical variables were expressed as frequencies and percentages, with intergroup differences assessed using the Chi-square test.

To examine the association between eGDR and cognitive decline, a linear mixed-effects model with random intercepts and slopes was used to assess changes in cognitive function over the follow-up period. The longitudinal relationship was modeled by including an interaction term between eGDR group and follow-up time. Since the mixed-effects model accounts for random missing data, no additional imputation procedures were applied (Li et al., 2021; Li C. et al., 2022). To ensure robustness and consistency, sensitivity analyses were performed by stepwise adjustment of covariates. Model 1 adjusted for baseline age, age^2^, and sex (not included in sex-stratified analyses). Model 2 further adjusted for educational level, marital status, area of residence, smoking status, alcohol consumption, BMI, and BMI^2^. Model 3 additionally controlled for CRP, hemoglobin, dyslipidemia, diabetes, heart disease, and stroke. Because modifications to the memory tests (particularly immediate and delayed word recall) were introduced in the 2018 wave of CHARLS, we conducted a sensitivity analysis using an equipercentile equating procedure to convert the 2018 word recall scores to the 2015 scale (Wu Y. et al., 2024). We then re-estimated our main linear mixed-effects models using these equated scores to address potential test-form bias.

The potential dose-response relationship between eGDR and cognitive decline was further explored by including both linear and quadratic terms of eGDR, along with their interaction with time, in the linear mixed-effects model (Model 3). The interaction term eGDR × time was used to estimate the effect of eGDR on the rate of cognitive decline.

All results are reported as β (95% confidence interval), where β represents the effect of a one-unit change in eGDR on the standardized *Z*-score change in cognitive function. Statistical analyses were conducted using R version 4.4.1,^1^ with a significance threshold set at *P* < 0.05.

## 3 Results

### 3.1 Baseline characteristics of participants

Baseline characteristics of the total population and sex-specific subgroups stratified by eGDR tertiles are shown in Table 1 and Supplementary Tables S1, S2, respectively. Among the 8,734 participants, the median age was (IQR, 52–65), including 4,056 males (46.4%) and 4,678 females (53.6%).

**TABLE 1:** Baseline characteristics of participants stratified by eGDR tertiles.

	Tertile 1	Tertile 2	Tertile 3	*P*
eGDR (mg/kg/min)	≤7.80	7.80–10.75	>10.75	
N	2,912	2,912	2,910	
Sex, *n* (%)	1,284 (44.1)	1,315 (45.2)	1,457 (50.1)	<0.001
Age (years)	60 [54, 67]	58 [51, 64]	57 [51, 63]	<0.001
Married or cohabiting, *n* (%)	2,519 (86.5)	2,586 (88.8)	2,616 (89.9)	<0.001
**Educational, *n* (%) ^a^**				0.005
Elementary school or below	2,082 (71.5)	1,989 (68.3)	2,055 (70.6)	
Secondary school	783 (26.9)	895 (30.7)	816 (28.1)	
College and above	47 (1.6)	28 (1.0)	38 (1.3)	
Rural, *n* (%)	1,753 (60.2)	1,904 (65.4)	2,102 (72.2)	<0.001
Smoking, *n* (%) ^a^	1,057 (36.3)	1,109 (38.1)	1,256 (43.2)	<0.001
Drinking, *n* (%)	657 (22.6)	747 (25.7)	793 (27.3)	<0.001
Hypertension, *n* (%)	2,876 (98.8)	718 (24.7)	10 (0.3)	<0.001
Dyslipidemia, *n* (%) ^a^	1,615 (55.6)	1,268 (43.6)	826 (28.4)	<0.001
Diabetes, *n* (%)	668 (22.9)	447 (15.4)	187 (6.4)	<0.001
Heart disease, *n* (%) ^a^	501 (17.3)	285 (9.8)	221 (7.6)	<0.001
Stroke, *n* (%)^a^	101 (3.5)	38 (1.3)	23 (0.8)	<0.001
BMI (kg/m^2^) ^a^	25.28 [23.08, 27.81]	23.97 [21.63, 26.22]	20.99 [19.49, 22.51]	<0.001
Waist (cm)	91.00 [86.00, 97.50]	88.00 [81.00, 93.00]	77.50 [73.40, 81.00]	<0.001
HBAlc (%)	5.20 [4.90, 5.60]	5.20 [4.90, 5.50]	5.00 [4.80, 5.30]	<0.001
CRP (mg/L) ^a^	1.37 [0.73, 2.73]	1.03 [0.58, 2.09]	0.75 [0.44, 1.58]	<0.001
Hemoglobin (g/dL)^a^	14.50 [13.30, 15.80]	14.30 [13.10, 15.60]	14.00 [12.80, 15.20]	<0.001
eGDR (mg/kg/min)	6.64 [5.99, 7.20]	9.82 [8.74, 10.36]	11.39 [11.06, 11.78]	<0.001
Cognitive function	11.50 [8.00, 14.00]	11.50 [8.00, 14.00]	11.00 [7.50, 14.00]	0.161

Data are shown as medians [interquartile ranges] or numbers (percentages). eGDR, estimated glucose disposal rate; BMI, body mass index; HbA1c, hemoglobin A1c; CRP, C-reactive protein.

*^a^*There were 1, 1, 17, 18, 38, 51, 118, and 175 participants who missed the measurement of educational, smoking, stroke, dyslipidemia, heart disease, BMI, CRP, and hemoglobin measurements, respectively.

Participants in the highest eGDR tertile were generally younger, had lower BMI and CRP, and exhibited a lower prevalence of dyslipidemia, diabetes, heart disease, and stroke compared to those in the lowest tertile.

### 3.2 Sex differences in eGDR and cognitive function

At baseline, females had significantly lower eGDR values [median: 9.69 (IQR, 7.10–11.00) vs. 9.98 (IQR, 7.34–11.13); *P* < 0.001] and baseline cognitive function scores [median: 10.0 (IQR, 6.5–13.5) vs. 12.5 (IQR, 9.5–14.5); *P* < 0.001] compared to males (Supplementary Figure S1). Although both sexes showed a bimodal distribution of eGDR in the overall population, stratification by hypertension status revealed a unimodal distribution in both groups (Supplementary Figure S2), suggesting that the bimodal pattern may be partially influenced by hypertension status. From 2011 to 2018, cognitive function scores declined across all groups, with males consistently displaying the highest scores and females the lowest across all domains—both before and after the equipercentile conversion (Supplementary Figure S3).

### 3.3 Association between eGDR tertiles and global cognitive decline

**FIGURE 2:**
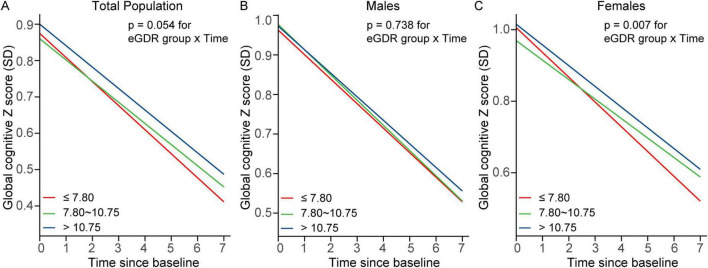
Trajectories of global cognitive Z scores by eGDR tertiles in **(A)** the total population, **(B)** males, and **(C)** females. Linear mixed-effects models with random intercepts and slopes were adjusted for covariates, including age, age^2^, educational level, marital status, residence, smoking status, drinking status, BMI, BMI^2^, C-reactive protein, hemoglobin, dyslipidemia, diabetes, heart disease, and stroke. Sex was included as an additional covariate in the total population model. BMI, body mass index; eGDR, estimated glucose disposal rate; SD, standard deviation.

Table 2 and Figure 2 show the associations between eGDR tertiles and global cognitive decline over the 7-year follow-up period, adjusted for covariates. In the total population, participants in Tertile 2 (β = 0.008; 95% CI: 0.001–0.015; *P* = 0.029) and Tertile 3 (β = 0.007; 95% CI: 0.000–0.014; *P* = 0.047) demonstrated significantly slower rates of global cognitive decline compared to those in Tertile 1.

**TABLE 2:** Associations between eGDR tertiles and annual change in global cognitive function *Z*-scores (SD/year) over 7 years of follow-up.

eGDR tertiles	Model 1		Model 2		Model 3	
	**(β, 95% CI)**	***P***	**(β, 95% CI)**	***P***	**(β, 95% CI)**	***P***
**Total population**						
Tertile 1	0 (Reference)		0 (Reference)		0 (Reference)	
Tertile 2	0.009 (0.002, 0.016)	0.009	0.009 (0.002, 0.016)	0.016	0.008 (0.001, 0.015)	0.029
Tertile 3	0.008 (0.001, 0.015)	0.028	0.007 (0.000, 0.014)	0.037	0.007 (0.000, 0.014)	0.047
Test for trend		0.014		0.020		0.016
**Males**						
Tertile 1	0 (Reference)		0 (Reference)		0 (Reference)	
Tertile 2	0.001 (−0.010, 0.011)	0.903	−0.000 (−0.010, 0.010)	0.987	−0.002 (−0.012, 0.009)	0.747
Tertile 3	0.003 (−0.007, 0.014)	0.518	0.003 (−0.007, 0.014)	0.531	0.002 (−0.008, 0.013)	0.652
Test for trend		0.562		0.596		0.489
**Females**						
Tertile 1	0 (Reference)		0 (Reference)		0 (Reference)	
Tertile 2	0.015 (0.006, 0.025)	0.001	0.015 (0.005, 0.024)	0.002	0.015 (0.005, 0.024)	0.003
Tertile 3	0.012 (0.002, 0.021)	0.014	0.011 (0.002, 0.021)	0.019	0.011 (0.002, 0.021)	0.021
Test for trend		0.005		0.008		0.009

Data were analyzed using linear mixed-effects regression models. The β values (95% CI) represent the annual change in cognitive *Z*-scores (SD/year) associated with eGDR, relative to the reference group (Tertile 1). eGDR, Estimated glucose disposal rate; β, Regression coefficient; CI, Confidence interval. Model 1: Adjusted for baseline age, age^2^, and sex (not included in sex-stratified analyses). Model 2: Further adjusted for educational level, marital status, residence, smoking status, drinking status, BMI, and BMI^2^. Model 3: Additionally adjusted for C-reactive protein, hemoglobin, dyslipidemia, diabetes, heart disease, and stroke.

In females, Tertile 2 was significantly associated with slower cognitive decline (β = 0.015; 95% CI: 0.005–0.024; *P* = 0.003), and this association persisted in Tertile 3 (β = 0.011; 95% CI: 0.002–0.021; *P* = 0.021). In contrast, no significant associations were observed between eGDR tertiles and cognitive decline in males.

As a sensitivity analysis, we applied an equipercentile equating procedure to the 2018 memory scores. Results remained largely consistent: in the total population, Tertile 2 (β = 0.007; 95% CI: 0.000–0.014; *P* = 0.036) and Tertile 3 (β = 0.007; 95% CI: 0.001–0.015; *P* = 0.033) were significantly linked to slower cognitive decline, and in females, Tertile 2 (β = 0.013; 95% CI: 0.004–0.023; *P* = 0.004) and Tertile 3 (β = 0.011; 95% CI: 0.002–0.020; *P* = 0.022) continued to show protective effects. No significant associations were detected among males (Supplementary Table S3).

### 3.4 Association between eGDR tertiles and executive function decline

As shown in Supplementary Figure S4 and Supplementary Table S4, in the total population, Tertile 3 (β = 0.008; 95% CI: 0.001–0.014; *P* = 0.020) exhibited significantly slower rates of executive function decline compared to those in Tertile 1. The association for Tertile 2 (β = 0.006; 95% CI: 0.000–0.013; *P* = 0.059) did not reach statistical significance but showed a similar trend.

In females, Tertile 2 was associated with a significantly slower rate of executive function decline (β = 0.012; 95% CI: 0.003–0.021; *P* = 0.006), and this association remained significant for Tertile 3 (β = 0.010; 95% CI: 0.001–0.019; *P* = 0.031). No significant associations were observed in males.

As a sensitivity analysis, we applied an equipercentile equating procedure to the 2018 memory scores. Under this approach, Tertile 2 (β = 0.006; 95% CI: 0.000–0.013; *P* = 0.060) and Tertile 3 (β = 0.008; 95% CI: 0.001–0.014; *P* = 0.021) in the total population again showed significantly slower decline compared to Tertile 1. In females, Tertile 2 (β = 0.012; 95% CI: 0.003–0.021; *P* = 0.006) and Tertile 3 (β = 0.010; 95% CI: 0.001–0.018; *P* = 0.031) remained significant, and no associations were noted in males (Supplementary Table S5).

### 3.5 Association between eGDR tertiles and episodic memory decline

In females, Tertile 2 was significantly associated with improved episodic memory scores compared to Tertile 1 (β = 0.013; 95% CI: 0.001–0.026; *P* = 0.040 in Model 1; β = 0.013; 95% CI: 0.000–0.026; *P* = 0.045 in Model 2). This association remained marginally significant in Model 3 (β = 0.013; 95% CI: 0.000–0.026; *p* = 0.050). No significant associations were observed in males or the total population (Supplementary Figure S5 and Supplementary Table S6).

As a sensitivity analysis, we applied an equipercentile equating procedure to the 2018 memory scores (Supplementary Table S7). Under this approach, none of the eGDR tertiles were significantly associated with episodic memory changes for the total population or males. Among females, Tertile 2 showed a borderline association (*P* = 0.096 in Model 3), but did not reach conventional levels of statistical significance.

### 3.6 Dose-response relationship between baseline eGDR and global cognitive decline

In the total population, higher baseline eGDR levels were significantly associated with slower global cognitive decline. The interaction between eGDR and time was significant (β = 0.002; 95% CI: 0.001–0.003; *P* = 0.002), indicating that for every 1-unit increase in eGDR, the annual decline in cognitive *Z*-scores slowed by 0.002 SD. The relationship between eGDR and cognitive decline was linear (*P* for nonlinearity = 0.609).

**FIGURE 3:**
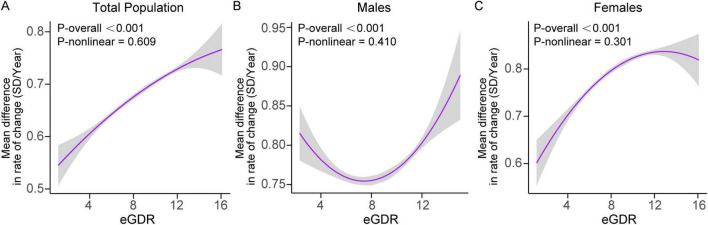
Dose-response curves of eGDR and the rate of global cognitive decline (SD/year) in **(A)** the total population, **(B)** males, and **(C)** females. Mixed linear regression models with random intercepts and slopes were adjusted for covariates, including age, age^2^, educational level, marital status, residence, smoking status, drinking status, BMI, BMI^2^, C-reactive protein, hemoglobin, dyslipidemia, diabetes, heart disease, and stroke. Sex was included as a covariate in the total population model. Shaded areas indicate 95% confidence intervals. BMI, body mass index; eGDR, estimated glucose disposal rate; SD, standard deviation.

In females, a significant interaction between eGDR and time (β = 0.003; 95% CI: 0.001–0.004; *P* = 0.003) confirmed that higher eGDR levels were associated with slower rates of cognitive decline over time (Figure 3 and Supplementary Table S8). Similarly, the dose-response relationship was linear (*P* for nonlinearity = 0.301). In males, no significant interaction was observed between eGDR and time (β = 0.001; 95% CI: -0.001–0.003; *P* = 0.187), suggesting no clear dose-response relationship in this subgroup.

### 3.7 Dose-response relationship between baseline eGDR and executive function decline

In the total population, a significant interaction between baseline eGDR and time was observed for executive function decline (β = 0.002; 95% CI: 0.001–0.003; *P* = 0.001), suggesting that higher eGDR values were associated with a slower rate of decline in executive function over time. This association was consistent across sexes. In males, higher eGDR values were significantly linked to slower declines in executive function (β = 0.002; 95% CI: 0.000–0.004; *P* = 0.032), while in females, the association was similarly significant (β = 0.002; 95% CI: 0.000–0.004; *P* = 0.011) (Supplementary Figure S6 and Supplementary Table S9). The dose-response relationship between eGDR and executive function decline appeared to follow a linear pattern (*P* for nonlinearity > 0.05).

### 3.8 Dose-response relationship between baseline eGDR and episodic memory decline

In females, a significant interaction between baseline eGDR and time was observed for episodic memory decline (β = 0.002; 95% CI: 0.000–0.005; *P* = 0.039), indicating that higher eGDR values were associated with a slower rate of decline in episodic memory over time (Supplementary Figure S7 and Supplementary Table S10). This relationship also appeared to be linear (*P* for nonlinearity = 0.578).

In contrast, no significant interactions were found in males (β = 0.000; 95% CI: -0.003–0.002; *P* = 0.839) or in the total population (β = 0.001; 95% CI: 0.000–0.003; *P* = 0.138).

### 3.9 Other analyses: covariates and their influence on cognitive decline

Supplementary Table S11 presents the associations between covariates and cognitive decline in the total population. Educational level emerged as a significant predictor of cognitive decline, with individuals with elementary school education or below exhibiting the most pronounced decline (standardized β = −0.953; 95% CI: -1.088 to 0.819; *P* < 0.001), followed by those with secondary school education (standardized β = V0.278; 95% CI: -0.412 to -0.143; *P* < 0.001). The inclusion of educational level in the model explained an additional 8.9% of the variance in cognitive decline (Δ*R*^2^ = 0.089), making it the most influential factor in the model, ahead of sex. After controlling for established risk factors such as age and educational level, sex remained a significant predictor of cognitive decline (standardized β = −0.180; 95% CI: -0.202 to -0.158; *P* < 0.001), contributing an additional 1.6% to the explained variance (Δ*R*^2^ = 0.016). This underscores the independent role of sex in explaining cognitive decline, independent of other established risk factors. Age demonstrated a nonlinear relationship with cognitive decline, with the quadratic term for age showing significant negative effects (standardized β = −0.615; 95% CI: -0.779 to -0.451; *P* < 0.001), suggesting that the rate of cognitive decline accelerates with increasing age, particularly among older individuals. Higher BMI was positively associated with cognitive performance (standardized β = 0.302; 95% CI: 0.222–0.381; *P* < 0.001), while its squared term indicated a nonlinear relationship, suggesting diminishing benefits at higher BMI levels (standardized β = −0.221; 95% CI: -0.298 to -0.145; *P* < 0.001).

## 4 Discussion

This study is the first large-scale longitudinal investigation to examine the association between eGDR and cognitive decline in middle-aged and older Chinese adults, with a particular focus on sex-specific differences. The findings reveal that higher eGDR levels are significantly associated with a slower rate of cognitive decline, but only in women, while no such association was observed in men. These results suggest that eGDR may exert a stronger protective effect on cognitive function in women, providing novel insights into the sex-specific impacts of IR on cognitive decline.

Although studies in Western populations have consistently shown an association between IR and cognitive decline (Fava et al., 2017; Hooshmand et al., 2019; Neergaard et al., 2017; Sherzai et al., 2018; Smith et al., 2019), evidence from Asian populations remains inconsistent (Ji et al., 2024b; Kong et al., 2018; Lee et al., 2019; Ma and Li, 2017; Yu et al., 2023). As a country that accounts for 30% of the global dementia burden (Wang et al., 2024), China is projected to face the highest economic impact of dementia in the future (Chen et al., 2024), highlighting the urgent need for large-scale longitudinal studies. Furthermore, research on sex differences in the relationship between IR and cognitive decline remains scarce. This study addresses these critical gaps by utilizing the CHARLS cohort, a nationally representative sample of middle-aged and older Chinese adults, thereby enhancing the generalizability of our findings to the broader Chinese population. Our results provide new insights into the role of eGDR as a surrogate marker for IR and its association with cognitive decline, with particular emphasis on sex-specific differences.

Baseline analysis revealed that eGDR levels and cognitive scores were both significantly lower in women than in men, consistent with previous findings on sex-specific differences in metabolic and cognitive characteristics. Postmenopausal women are particularly susceptible to reduced insulin sensitivity due to declining estrogen levels (Ciarambino et al., 2023). Furthermore, studies across regions have shown that cognitive scores in Chinese women remain significantly lower than in men, even after accounting for educational differences (Bloomberg et al., 2023). Moreover, sex continued to show significant additional explanatory power for cognitive decline (Δ*R*^2^ = 0.016) after controlling for other established risk factors, including age and educational level, indicating that sex provides an independent contribution to cognitive decline. This finding supports the hypothesis that sex-specific metabolic mechanisms, such as hormonal influences or IR, may influence cognitive function, particularly in women, where IR may accelerate cognitive decline through distinct metabolic pathways. These findings emphasize the importance of sex-stratified analyses to better understand the relationship between eGDR and cognitive decline.

Prior research has highlighted sex differences in the association between IR and cognitive decline. For example, a study of non-diabetic older adults in the United States found that higher TyG index levels were significantly associated with lower cognitive function in women, whereas no such association was observed in men (Wei et al., 2023). Similarly, a nationwide study in Finland reported that higher HOMA-IR levels were associated with poorer verbal fluency in women but not in men (Ekblad et al., 2015). These findings suggest that IR may exert a more pronounced impact on cognitive function in women. Our findings align with these observations, showing that lower eGDR levels may serve as an early predictor of cognitive decline in women, while no such association was observed in men. This highlights the need for sex-specific strategies in addressing the cognitive impacts of IR.

Sex differences in the relationship between IR and cognitive decline are likely influenced by a combination of physiological, metabolic, and social factors. Estrogen has demonstrated neuroprotective effects, likely through mechanisms such as modulation of neurotransmission, enhancement of cerebral blood flow, and promotion of neuronal repair (Genazzani et al., 2007). However, following menopause, women experience a significant decline in estrogen levels, which reduces its neuroprotective impact (Genazzani et al., 2007). As a result, the negative association between eGDR and cognitive decline may be more pronounced in women. Similarly, testosterone also exerts neuroprotective effects, potentially mitigating cognitive decline by slowing neuronal death and promoting neurorepair mechanisms (Bianchi, 2022). Additionally, the loss of testosterone with aging has been linked to an increased risk of dementia (Ahmadpour and Grange-Messent, 2021; Moffat and Resnick, 2007). While androgen levels in men gradually decline with age, this decline typically occurs more slowly, which may mask the relationship between eGDR and cognitive decline in the male population. Moreover, immune response differences between men and women may also contribute (Klein and Flanagan, 2016). Women generally exhibit stronger innate and adaptive immune responses, which can amplify inflammatory pathways and worsen the impact of IR on cognitive function (Klein and Flanagan, 2016; Zhou et al., 2024). In addition, while eGDR serves as a practical, non-invasive marker of insulin sensitivity, it also reflects broader cardiovascular and metabolic risks, as it incorporates waist circumference, hypertension, and HbA1c. Previous studies in China have shown that education level may significantly modify the relationship between cardiometabolic biomarkers and cognitive outcomes (Srikantha et al., 2024; Wu Q. et al., 2024). For example, diabetes was inversely related to average cognitive scores among individuals with less than lower secondary education (Srikantha et al., 2024), while in the highest-educated elderly population, higher cardiometabolic risk was associated with better cognitive function and slower cognitive decline (Wu Q, et al., 2024). These findings underscore the importance of considering social factors, such as education level, when interpreting the relationship between insulin resistance and cognitive decline, particularly in large-scale longitudinal studies. Our baseline data indicate that women have significantly lower levels of education than men; approximately 40% of males have attended secondary school or higher, while only around 20% of females have achieved the same educational level (Supplementary Table S12). This educational disparity may help explain why a significant association between eGDR and cognitive decline was observed in women but not in men.

This study has several limitations. First, although adjustments were made for several covariates, such as age, sex, and education level, unmeasured confounders, including dietary and genetic factors (Dissanayaka et al., 2024; Ji et al., 2024a), may still influence the observed associations. Second, as a longitudinal observational study, causality cannot be established. Nonetheless, the consistency of our findings across multiple analytical models and their alignment with existing mechanistic studies suggest that these associations are unlikely to be entirely due to confounding. Future research should explore genetic predispositions, lifestyle factors, and dietary habits to provide a more comprehensive understanding of the role of IR in cognitive decline.

## 5 Conclusion

This study identified a significant association between higher eGDR levels and slower cognitive decline among middle-aged and older adults in China, with a more pronounced effect observed in women. These findings suggest that eGDR could serve as a potential marker for assessing the risk of cognitive decline and underscore the importance of further research into sex-specific mechanisms and targeted interventions aimed at improving IR and cognitive health.

## Data Availability

The original contributions presented in the study are included in the article/Supplementary material, further inquiries can be directed to the corresponding author.

## Footnotes

1
http://www.R-project.org/
