# Supplementary material for: Sex-specific associations between estimated glucose disposal rate and cognitive decline in middle-aged and older adults in China: a longitudinal cohort study
Source: Front Aging Neurosci. 2025 Feb 5;17:1544352. doi: 10.3389/fnagi.2025.1544352 (PMC11835878; doi:10.3389/fnagi.2025.1544352)
Supplement: Supplementary file 1 [file Table_1.docx]

Sex-Specific Associations Between Estimated Glucose Disposal Rate and Cognitive Decline in Middle-Aged and Older Adults in China: A Longitudinal Cohort Study

Supplementary Material

## Supplementary Figures


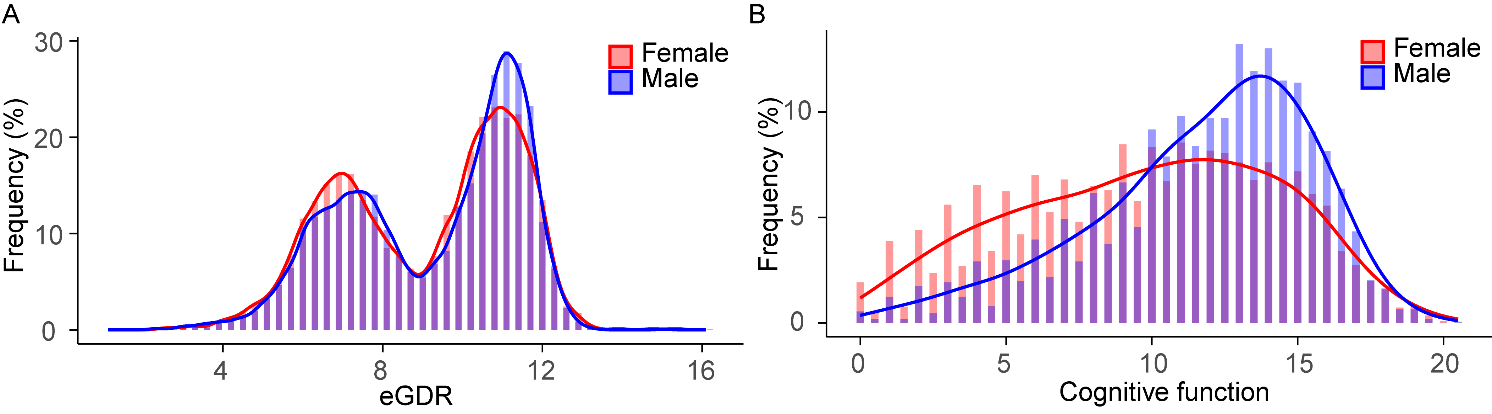


**Supplementary Figure 1.** Baseline distributions of eGDR and cognitive function by sex. The eGDR levels show a bimodal distribution, with females generally concentrated at lower values and males at higher values. Cognitive function scores are more clustered at lower values in females and higher values in males. eGDR: estimated glucose disposal rate.


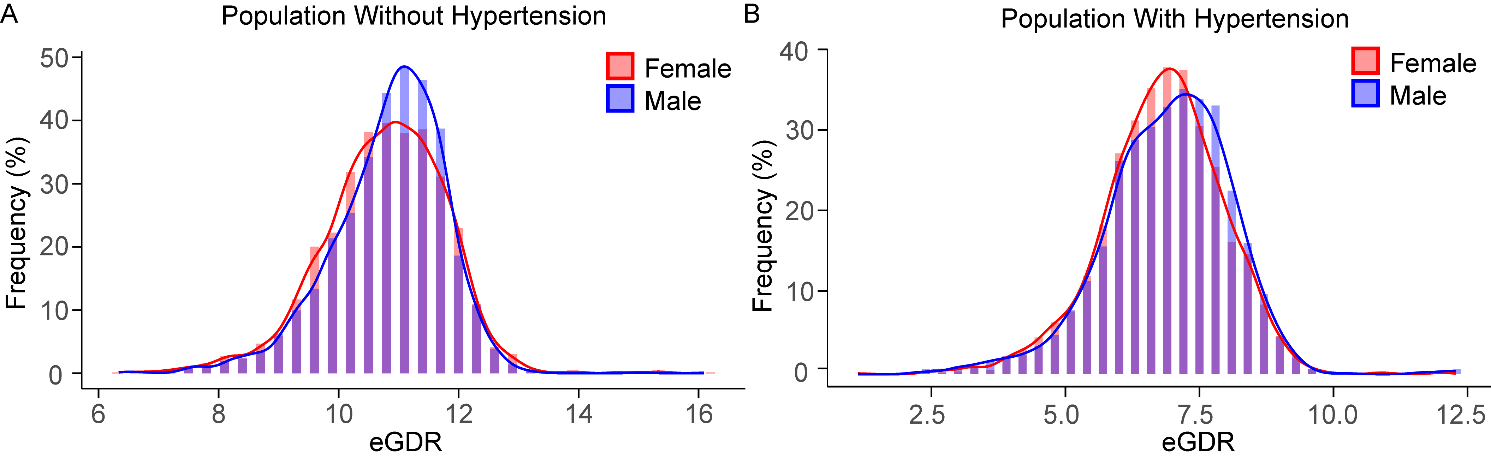


**Supplementary Figure 2.** Baseline distributions of eGDR by hypertension status. (A) eGDR distribution among males and females without hypertension at baseline. (B) eGDR distribution among males and females with hypertension at baseline. eGDR: estimated glucose disposal rate.


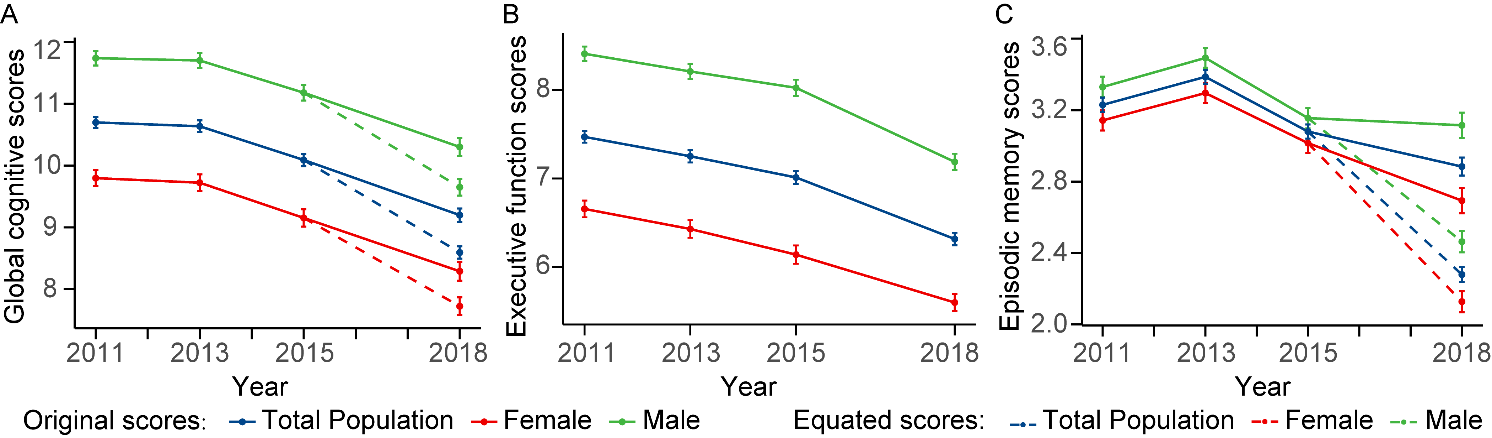


**Supplementary Figure 3.** Trends in average cognitive function scores across different groups (total population, male, and female) from 2011 to 2018, comparing original and equated scores. (A) Global cognitive function scores. (B) Executive function scores. (C) Episodic memory scores. Solid lines represent original scores, and dashed lines represent equated scores. Error bars indicate standard errors.


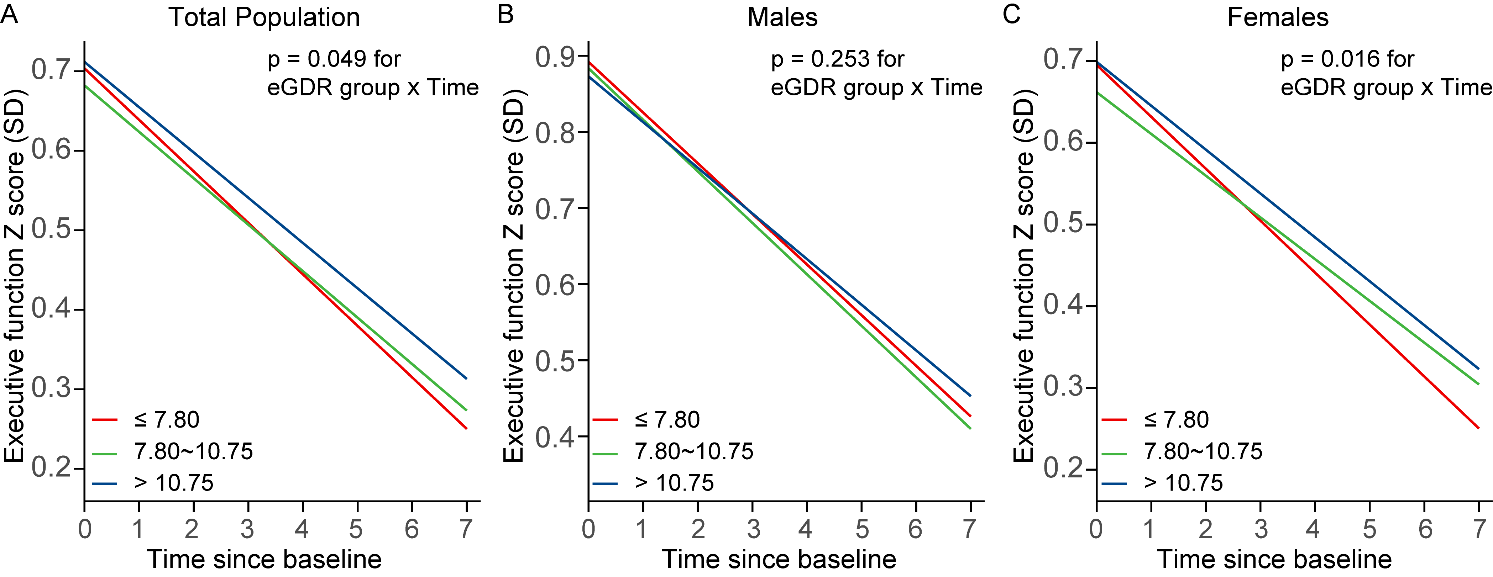


**Supplementary Figure 4.** Trajectories of executive function Z scores over follow-up by eGDR tertiles in (A) the total population, (B) males, and (C) females. Trajectories are modeled using linear mixed-effects regression models with random intercepts and slopes, adjusted for age, age squared, educational level, marital status, residence, smoking status, drinking status, BMI, BMI squared, C-reactive protein, hemoglobin, dyslipidemia, diabetes, heart disease, and stroke. For the total population model, sex was also included as a covariate. BMI, body mass index; eGDR, estimated glucose disposal rate; SD, standard deviation.


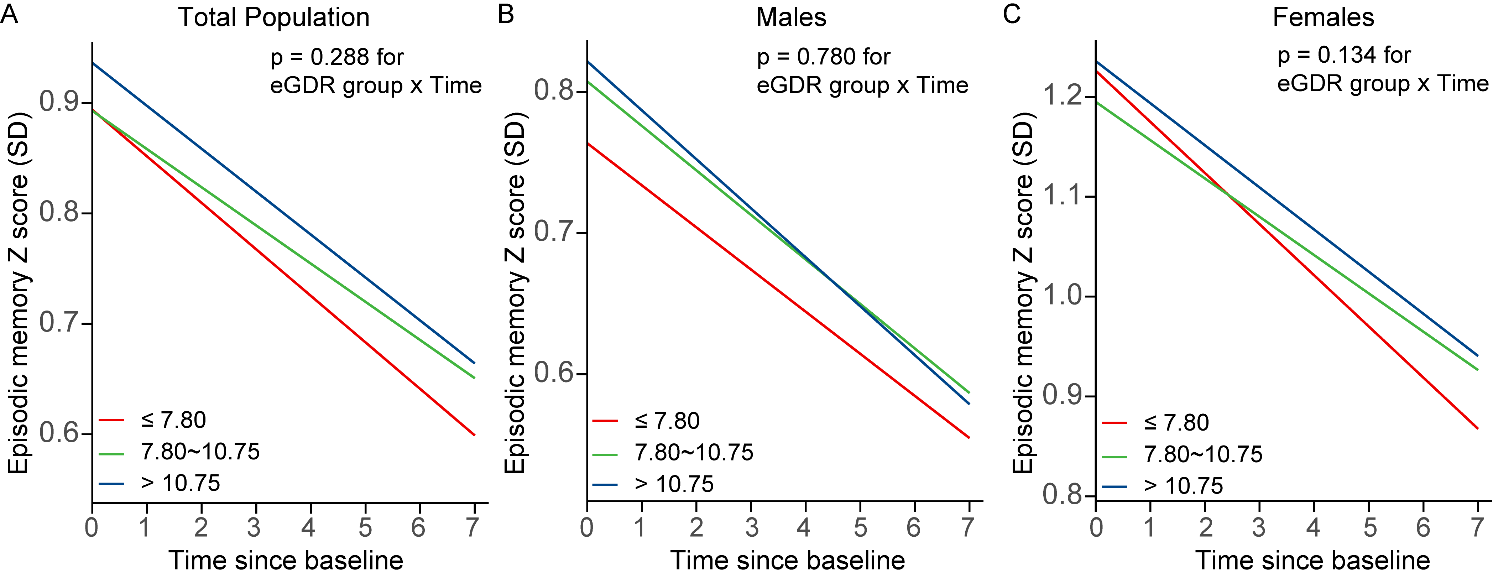


**Supplementary Figure 5.** Trajectories of episodic memory Z scores over follow-up by eGDR tertiles in the total population, males, and females. Trajectories are modeled using linear mixed-effects regression models with random intercepts and slopes, adjusted for age, age squared, educational level, marital status, residence, smoking status, drinking status, BMI, BMI squared, C-reactive protein, hemoglobin, dyslipidemia, diabetes, heart disease, and stroke. For the total population model, sex was also included as a covariate. BMI, body mass index; eGDR, estimated glucose disposal rate; SD, standard deviation.


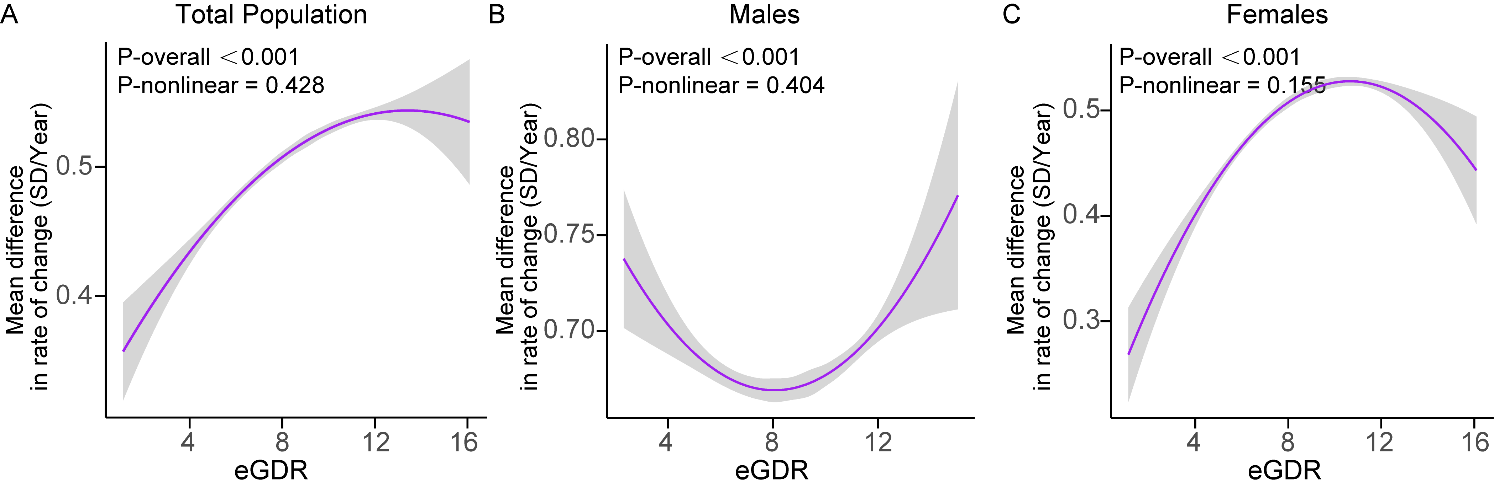


**Supplementary Figure 6**. Dose-response curves of eGDR and rate of executive function decline (SD/Year) in the total population, males, and females. Mixed linear regression models with random intercepts and slopes were adjusted for age, age2, educational level, marital status, residence, smoking status, drinking status, BMI, BMI2, C-reactive protein, hemoglobin, dyslipidemia, diabetes, heart disease, and stroke. For the total population model, sex was also included as a covariate. Shaded areas represent 95% confidence intervals. eGDR, estimated glucose disposal rate; BMI, body mass index; SD, standard deviation.


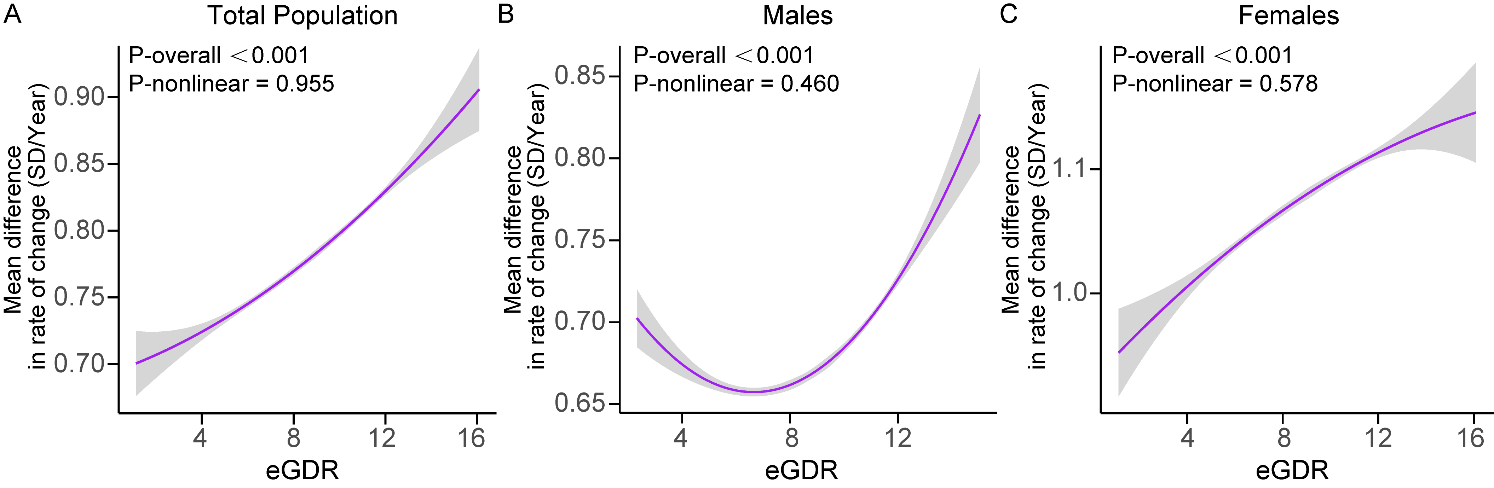


**Supplementary Figure 7**. Dose-response curves of eGDR and rate of episodic memory decline (SD/Year) in the total population, males, and females. Mixed linear regression models with random intercepts and slopes were adjusted for age, age2, educational level, marital status, residence, smoking status, drinking status, BMI, BMI2, C-reactive protein, hemoglobin, dyslipidemia, diabetes, heart disease, and stroke. For the total population model, sex was also included as a covariate. Shaded areas represent 95% confidence intervals. eGDR, estimated glucose disposal rate; BMI, body mass index; SD, standard deviation.

**Supplementary Tables**

**Supplementary Table 1. Baseline Characteristics of Male Participants**

|  | Tertile 1 | Tertile 2 | Tertile 3 | P |
| --- | --- | --- | --- | --- |
| eGDR (mg/kg/min) | ≤7.92 | 7.92-10.85 | >10.85 |  |
| n | 1352 | 1352 | 1352 |  |
| Age (years) | 61 [54, 67] | 58 [52, 65] | 59 [53, 65] | <0.001 |
| Married or Cohabiting, n (%) | 1,234 (91.3) | 1,237 (91.5) | 1,228 (90.8) | 0.824 |
| Educational, n (%) ^a^ |  |  |  | <0.001 |
| Elementary school or below | 803 (59.4) | 779 (57.6) | 884 (65.4) |  |
| Secondary school | 511 (37.8) | 548 (40.5) | 452 (33.5) |  |
| College and above | 38 (2.8) | 25 (1.8) | 15 (1.1) |  |
| Rural, n (%) | 830 (61.4) | 892 (66.0) | 1015 (75.1) | <0.001 |
| Smoking, n (%) | 975 (72.1) | 1014 (75.0) | 1063 (78.6) | <0.001 |
| Drinking, n (%) | 600 (44.4) | 631 (46.7) | 634 (46.9) | 0.348 |
| Hypertension, n (%) | 1,335 (98.7) | 296 (21.9) | 6 (0.4) | <0.001 |
| Dyslipidemia, n (%) ^a^ | 716 (53.2) | 596 (44.2) | 375 (27.8) | <0.001 |
| Diabetes, n (%) | 298 (22.0) | 194 (14.3) | 87 (6.4) | <0.001 |
| Heart disease, n (%) ^a^ | 210 (15.6) | 111 (8.2) | 88 (6.5) | <0.001 |
| Stroke, n (%) ^a^ | 50 (3.7) | 22 (1.6) | 9 (0.7) | <0.001 |
| BMI (kg/m^2^) ^a^ | 24.64 [22.53, 27.11] | 23.39 [21.16, 25.42] | 20.56 [19.30, 21.92] | <0.001 |
| Waist (cm) | 90.40 [84.60, 97.20] | 87.00 [81.95, 92.60] | 77.10 [73.50, 80.00] | <0.001 |
| HBAlc (%) | 5.20 [4.90, 5.50] | 5.20 [4.90, 5.50] | 5.00 [4.80, 5.30] | <0.001 |
| CRP (mg/L) ^a^ | 1.34 [0.70, 2.59] | 1.07 [0.59, 2.17] | 0.82 [0.48, 1.85] | <0.001 |
| Hemoglobin (g/dL) ^a^ | 15.30 [14.20, 16.60] | 15.10 [14.08, 16.30] | 14.80 [13.70, 15.90] | <0.001 |
| eGDR (mg/kg/min) | 6.73 [6.05, 7.34] | 9.98 [8.98, 10.50] | 11.42 [11.13, 11.76] | <0.001 |
| Cognitive function | 12.50 [10.00, 14.50] | 12.50 [10.00, 14.50] | 12.00 [9.00, 14.00] | <0.001 |

Data are shown as medians [interquartile ranges] or numbers (percentages).

Abbreviations: eGDR, estimated glucose disposal rate; BMI, body mass index; HbA1c, hemoglobin A1c; CRP, C-reactive protein.

^a^ There were 1, 11, 11, 17, 21, 55 and 77 participants who missed the measurement of educational, stroke, dyslipidemia, heart disease, BMI, CRP and hemoglobin measurements, respectively

**Supplementary Table 2. Baseline Characteristics of Female Participants**

|  | Tertile 1 | Tertile 2 | Tertile 3 | P |
| --- | --- | --- | --- | --- |
| eGDR (mg/kg/min) | ≤7.66 | 7.66-10.64 | >10.64 |  |
| n | 1561 | 1559 | 1558 |  |
| Age (years) | 60 [54, 67] | 58 [50, 64] | 55 [49, 61] | <0.001 |
| Married or cohabiting, n (%) | 1,296 (83.0) | 1,338 (85.8) | 1,388 (89.1) | <0.001 |
| Educational, n (%) |  |  |  | <0.001 |
| Elementary school or below | 1,267 (81.2) | 1,199 (76.9) | 1,194 (76.6) |  |
| Secondary school | 286 (18.3) | 354 (22.7) | 343 (22.0) |  |
| College and above | 8 (0.5) | 6 (0.4) | 21 (1.3) |  |
| Rural, n (%) | 927 (59.4) | 1,008 (64.7) | 1,087 (69.8) | <0.001 |
| Smoking, n (%) ^a^ | 132 (8.5) | 124 (8.0) | 114 (7.3) | 0.498 |
| Drinking, n (%) | 88 (5.6) | 119 (7.6) | 125 (8.0) | 0.021 |
| Hypertension, n (%) | 1543 (98.8) | 420 (26.9) | 4 (0.3) | <0.001 |
| Dyslipidemia, n (%) ^a^ | 897 (57.6) | 664 (42.6) | 461 (29.6) | <0.001 |
| Diabetes, n (%) | 375 (24.0) | 248 (15.9) | 100 (6.4) | <0.001 |
| Heart disease, n (%) ^a^ | 293 (18.9) | 174 (11.2) | 131 (8.4) | <0.001 |
| Stroke, n (%) ^a^ | 50 (3.2) | 17 (1.1) | 14 (0.9) | <0.001 |
| BMI (kg/m^2^) ^a^ | 25.80 [23.50, 28.36] | 24.50 [22.07, 26.84] | 21.41 [19.74, 23.16] | <0.001 |
| Waist (cm) | 91.50 [86.80, 97.80] | 88.80 [80.15, 93.30] | 78.00 [73.20, 81.80] | <0.001 |
| HBAlc (%) | 5.30 [5.00, 5.60] | 5.20 [4.90, 5.50] | 5.00 [4.80, 5.30] | <0.001 |
| CRP (mg/L) ^a^ | 1.42 [0.76, 2.83] | 0.98 [0.56, 1.88] | 0.70 [0.42, 1.43] | <0.001 |
| Hemoglobin (g/dL) ^a^ | 13.90 [12.90, 14.90] | 13.60 [12.60, 14.60] | 13.30 [12.30, 14.30] | <0.001 |
| eGDR (mg/kg/min) | 6.57 [5.95, 7.10] | 9.69 [8.57, 10.25] | 11.37 [11.00, 11.80] | <0.001 |
| Cognitive function | 10.00 [6.00, 13.50] | 10.00 [6.50, 13.50] | 10.00 [6.50, 13.50] | 0.811 |

Data are shown as medians [interquartile ranges] or numbers (percentages).

Abbreviations: eGDR, estimated glucose disposal rate; BMI, body mass index; HbA1c, hemoglobin A1c, CRP, C-reactive protein.

^a^ There were 1, 6, 7, 21, 30, 63 and 98 participants who missed the measurement of smoking, stroke, dyslipidemia, heart disease, BMI, CRP and hemoglobin measurements, respectively.

**Supplementary Table 3. Associations between eGDR Tertiles and Annual Change in Global Cognitive Function Z-Scores (SD/year) After Equipercentile Equating of the 2018 Memory Scores**

| eGDR Tertiles | Model 1 | | Model 2 |  | Model 3 |  |
| --- | --- | --- | --- | --- | --- | --- |
|  | (β, 95% CI) | *P* | (β, 95% CI) | *P* | (β, 95% CI) | *P* |
| **Total population** |  |  |  |  |  |  |
| Tertile 1 | 0 (Reference) |  | 0 (Reference) |  | 0 (Reference) |  |
| Tertile 2 | 0.008 (0.002, 0.015) | 0.013 | 0.008 (0.001, 0.014) | 0.022 | 0.007 (0.000, 0.014) | 0.036 |
| Tertile 3 | 0.008 (0.001, 0.014) | 0.021 | 0.007 (0.001, 0.014) | 0.029 | 0.007 (0.001, 0.015) | 0.033 |
| Test for trend |  | 0.011 |  | 0.017 |  | 0.013 |
| **Males** |  |  |  |  |  |  |
| Tertile 1 | 0 (Reference) |  | 0 (Reference) |  | 0 (Reference) |  |
| Tertile 2 | 0.000 (-0.010, 0.010) | 0.946 | -0.000 (-0.010, 0.010) | 0.947 | -0.001 (-0.011, 0.008) | 0.718 |
| Tertile 3 | 0.004 (-0.005, 0.014) | 0.372 | 0.004 (-0.006, 0.014) | 0.400 | 0.004 (-0.006, 0.014) | 0.483 |
| Test for trend |  | 0.438 |  | 0.480 |  | 0.379 |
| **Females** |  |  |  |  |  |  |
| Tertile 1 | 0 (Reference) |  | 0 (Reference) |  | 0 (Reference) |  |
| Tertile 2 | 0.014 (0.005, 0.023) | 0.002 | 0.013 (0.004, 0.022) | 0.003 | 0.013 (0.004, 0.023) | 0.004 |
| Tertile 3 | 0.010 (0.002, 0.020) | 0.016 | 0.010 (0.001, 0.019) | 0.022 | 0.011 (0.002, 0.020) | 0.022 |
| Test for trend |  | 0.007 |  | 0.010 |  | 0.011 |

Data were analyzed using linear mixed-effects regression models. The β values (95% CI) represent the annual change in cognitive Z-scores (SD/year) associated with eGDR, relative to the reference group (Tertile 1).

Abbreviations: eGDR, Estimated glucose disposal rate; β, Regression coefficient; CI, Confidence interval.

Model 1: Adjusted for baseline age, age^2^, and sex (not included in sex-stratified analyses).

Model 2: Further adjusted for educational level, marital status, residence, smoking status, drinking status, BMI, and BMI^2^.

Model 3: Additionally adjusted for C-reactive protein, hemoglobin, dyslipidemia, diabetes, heart disease, and stroke.

**Supplementary Table 4. Association Between eGDR Tertiles and Rate of executive function Decline (SD/Year) Over 7 Years of Follow-Up Using Linear Mixed-Effects Regression**

| **eGDR Tertiles** | **Model 1** | | **Model 2** |  | **Model 3** |  |
| --- | --- | --- | --- | --- | --- | --- |
|  | (β, 95% CI) | ***P*** | (β, 95% CI) | ***P*** | (β, 95% CI) | ***P*** |
| **Total population** |  |  |  |  |  |  |
| Tertile 1 | 0 (Reference) |  | 0 (Reference) |  | 0 (Reference) |  |
| Tertile 2 | 0.008 (0.001, 0.014) | 0.023 | 0.007 (0.001, 0.014) | 0.032 | 0.006 (0.000, 0.013) | 0.059 |
| Tertile 3 | 0.008 (0.002, 0.015) | 0.015 | 0.008 (0.001, 0.014) | 0.021 | 0.008 (0.001, 0.014) | 0.020 |
| Test for trend |  | 0.009 |  | 0.014 |  | 0.009 |
| **Males** |  |  |  |  |  |  |
| Tertile 1 | 0 (Reference) |  | 0 (Reference) |  | 0 (Reference) |  |
| Tertile 2 | 0.001 (-0.008, 0.011) | 0.794 | 0.001 (-0.009, 0.011) | 0.861 | -0.001 (-0.011, 0.009) | 0.824 |
| Tertile 3 | 0.007 (-0.002, 0.017) | 0.143 | 0.007 (-0.003, 0.017) | 0.158 | 0.007 (-0.003, 0.016) | 0.192 |
| Test for trend |  | 0.190 |  | 0.212 |  | 0.167 |
| **Females** |  |  |  |  |  |  |
| Tertile 1 | 0 (Reference) |  | 0 (Reference) |  | 0 (Reference) |  |
| Tertile 2 | 0.013 (0.004, 0.022) | 0.004 | 0.012 (0.004, 0.021) | 0.006 | 0.012 (0.003, 0.021) | 0.006 |
| Tertile 3 | 0.01 (0.001, 0.019) | 0.027 | 0.009 (0.001, 0.018) | 0.035 | 0.010 (0.001, 0.019) | 0.031 |
| Test for trend |  | 0.013 |  | 0.017 |  | 0.016 |

Data were analyzed using linear mixed-effects regression models. The β values (95% CI) represent the annual rate of decline in cognitive Z-scores (SD/year) relative to the reference group (Tertile 1).

Abbreviations: eGDR, Estimated glucose disposal rate; β, Regression coefficient; CI, Confidence interval.

Model 1: Adjusted for baseline age, age^2^, and sex (not included in sex-stratified analyses).

Model 2: Further adjusted for educational level, marital status, residence, smoking status, drinking status, BMI, and BMI^2^.

Model 3: Additionally adjusted for C-reactive protein, hemoglobin, dyslipidemia, diabetes, heart disease, and stroke.

**Supplementary Table 5. Association Between eGDR Tertiles and Rate of Executive Function Decline (SD/Year) After Equipercentile Equating of the 2018 Memory Scores**

| **eGDR Tertiles** | **Model 1** | | **Model 2** |  | **Model 3** |  |
| --- | --- | --- | --- | --- | --- | --- |
|  | (β, 95% CI) | ***P*** | (β, 95% CI) | ***P*** | (β, 95% CI) | ***P*** |
| **Total population** |  |  |  |  |  |  |
| Tertile 1 | 0 (Reference) |  | 0 (Reference) |  | 0 (Reference) |  |
| Tertile 2 | 0.008 (0.001, 0.014) | 0.023 | 0.007 (0.001, 0.014) | 0.032 | 0.006 (0.000, 0.013) | 0.060 |
| Tertile 3 | 0.008 (0.002, 0.015) | 0.015 | 0.008 (0.001, 0.014) | 0.021 | 0.008 (0.001, 0.014) | 0.021 |
| Test for trend |  | 0.009 |  | 0.014 |  | 0.009 |
| **Males** |  |  |  |  |  |  |
| Tertile 1 | 0 (Reference) |  | 0 (Reference) |  | 0 (Reference) |  |
| Tertile 2 | 0.001 (-0.008, 0.011) | 0.794 | 0.001 (-0.009, 0.011) | 0.861 | -0.001 (-0.011, 0.009) | 0.824 |
| Tertile 3 | 0.007 (-0.002, 0.017) | 0.143 | 0.007 (-0.003, 0.017) | 0.158 | 0.007 (-0.003, 0.016) | 0.192 |
| Test for trend |  | 0.190 |  | 0.211 |  | 0.167 |
| **Females** |  |  |  |  |  |  |
| Tertile 1 | 0 (Reference) |  | 0 (Reference) |  | 0 (Reference) |  |
| Tertile 2 | 0.013 (0.004, 0.022) | 0.004 | 0.012 (0.004, 0.021) | 0.006 | 0.012 (0.003, 0.021) | 0.006 |
| Tertile 3 | 0.01 (0.001, 0.019) | 0.027 | 0.009 (0.001, 0.018) | 0.036 | 0.010 (0.001, 0.018) | 0.031 |
| Test for trend |  | 0.013 |  | 0.017 |  | 0.016 |

Data were analyzed using linear mixed-effects regression models. The β values (95% CI) represent the annual rate of decline in cognitive Z-scores (SD/year) relative to the reference group (Tertile 1).

Abbreviations: eGDR, Estimated glucose disposal rate; β, Regression coefficient; CI, Confidence interval.

Model 1: Adjusted for baseline age, age^2^, and sex (not included in sex-stratified analyses).

Model 2: Further adjusted for educational level, marital status, residence, smoking status, drinking status, BMI, and BMI^2^.

Model 3: Additionally adjusted for C-reactive protein, hemoglobin, dyslipidemia, diabetes, heart disease, and stroke.

**Supplementary Table 6. Associations between eGDR Tertiles and Annual Change in Episodic Memory Z-Scores (SD/year) Over 7 Years of Follow-Up**

| **eGDR Tertiles** | **Model 1** | | **Model 2** |  | **Model 3** |  |
| --- | --- | --- | --- | --- | --- | --- |
|  | (β, 95% CI) | ***P*** | (β, 95% CI) | ***P*** | (β, 95% CI) | ***P*** |
| **Total population** |  |  |  |  |  |  |
| Tertile 1 | 0 (Reference) |  | 0 (Reference) |  | 0 (Reference) |  |
| Tertile 2 | 0.008 (-0.001, 0.018) | 0.073 | 0.008 (-0.002, 0.017) | 0.100 | 0.008 (-0.002, 0.017) | 0.116 |
| Tertile 3 | 0.004 (-0.005, 0.014) | 0.343 | 0.004 (-0.005, 0.013) | 0.398 | 0.003 (-0.006, 0.013) | 0.498 |
| Test for trend |  | 0.230 |  | 0.275 |  | 0.285 |
| **Males** |  |  |  |  |  |  |
| Tertile 1 | 0 (Reference) |  | 0 (Reference) |  | 0 (Reference) |  |
| Tertile 2 | -0.001 (-0.014, 0.013) | 0.942 | -0.001 (-0.015, 0.012) | 0.843 | -0.002 (-0.015, 0.012) | 0.809 |
| Tertile 3 | -0.004 (-0.017, 0.01) | 0.606 | -0.004 (-0.017, 0.01) | 0.586 | -0.005 (-0.019, 0.009) | 0.488 |
| Test for trend |  | 0.643 |  | 0.611 |  | 0.717 |
| **Females** |  |  |  |  |  |  |
| Tertile 1 | 0 (Reference) |  | 0 (Reference) |  | 0 (Reference) |  |
| Tertile 2 | 0.013 (0.001, 0.026) | 0.040 | 0.013 (0.000, 0.026) | 0.045 | 0.013 (0.000, 0.026) | 0.050 |
| Tertile 3 | 0.010 (-0.003, 0.023) | 0.117 | 0.010 (-0.003, 0.022) | 0.135 | 0.009 (-0.004, 0.022) | 0.168 |
| Test for trend |  | 0.077 |  | 0.089 |  | 0.119 |

Data were analyzed using linear mixed-effects regression models. The β values (95% CI) represent the annual rate of decline in cognitive Z-scores (SD/year) relative to the reference group (Tertile 1).

Abbreviations: eGDR, Estimated glucose disposal rate; β, Regression coefficient; CI, Confidence interval.

Model 1: Adjusted for baseline age, age^2^, and sex (not included in sex-stratified analyses).

Model 2: Further adjusted for educational level, marital status, residence, smoking status, drinking status, BMI, and BMI^2^.

Model 3: Additionally adjusted for C-reactive protein, hemoglobin, dyslipidemia, diabetes, heart disease, and stroke.

**Supplementary Table 7. Associations between eGDR Tertiles and Annual Change in Episodic Memory Z-Scores (SD/year) Over 7 Years of Follow-Up After Equipercentile Equating of the 2018 Memory Scores**

| **eGDR Tertiles** | **Model 1** | | **Model 2** |  | **Model 3** |  |
| --- | --- | --- | --- | --- | --- | --- |
|  | (β, 95% CI) | ***P*** | (β, 95% CI) | ***P*** | (β, 95% CI) | ***P*** |
| **Total population** |  |  |  |  |  |  |
| Tertile 1 | 0 (Reference) |  | 0 (Reference) |  | 0 (Reference) |  |
| Tertile 2 | 0.006 (-0.002, 0.015) | 0.136 | 0.005 (-0.003, 0.014) | 0.180 | 0.006 (-0.003, 0.014) | 0.180 |
| Tertile 3 | 0.004 (-0.004, 0.013) | 0.286 | 0.004 (-0.004, 0.012) | 0.352 | 0.004 (-0.005, 0.012) | 0.402 |
| Test for trend |  | 0.216 |  | 0.271 |  | 0.272 |
| **Males** |  |  |  |  |  |  |
| Tertile 1 | 0 (Reference) |  | 0 (Reference) |  | 0 (Reference) |  |
| Tertile 2 | -0.001 (-0.013, 0.011) | 0.835 | -0.002 (-0.014, 0.010) | 0.737 | -0.002 (-0.014, 0.010) | 0.740 |
| Tertile 3 | -0.001 (-0.013, 0.011) | 0.857 | -0.002 (-0.014, 0.011) | 0.802 | -0.002 (-0.014, 0.010) | 0.729 |
| Test for trend |  | 0.830 |  | 0.766 |  | 0.893 |
| **Females** |  |  |  |  |  |  |
| Tertile 1 | 0 (Reference) |  | 0 (Reference) |  | 0 (Reference) |  |
| Tertile 2 | 0.010 (-0.002, 0.021) | 0.090 | 0.009 (-0.002, 0.021) | 0.099 | 0.010 (-0.002, 0.021) | 0.096 |
| Tertile 3 | 0.008 (-0.003, 0.020) | 0.151 | 0.008 (-0.003, 0.019) | 0.174 | 0.008 (-0.004, 0.019) | 0.191 |
| Test for trend |  | 0.113 |  | 0.129 |  | 0.167 |

Data were analyzed using linear mixed-effects regression models. The β values (95% CI) represent the annual rate of decline in cognitive Z-scores (SD/year) relative to the reference group (Tertile 1).

Abbreviations: eGDR, Estimated glucose disposal rate; β, Regression coefficient; CI, Confidence interval.

Model 1: Adjusted for baseline age, age^2^, and sex (not included in sex-stratified analyses).

Model 2: Further adjusted for educational level, marital status, residence, smoking status, drinking status, BMI, and BMI^2^.

Model 3: Additionally adjusted for C-reactive protein, hemoglobin, dyslipidemia, diabetes, heart disease, and stroke.

**Supplementary Table 8. Associations Between Continuous eGDR and Annual Decline in Global Cognitive Over 7 Years**

|  | **Model 1** | | **Model 2** |  | **Model 3** |  |
| --- | --- | --- | --- | --- | --- | --- |
|  | (β, 95% CI) | ***P*** | (β, 95% CI) | ***P*** | (β, 95% CI) | ***P*** |
| **Total population** | 0.002 (0.001, 0.004) | <0.001 | 0.002 (0.001, 0.004) | <0.001 | 0.002 (0.001, 0.003) | 0.002 |
| **Males** | 0.002 (0.000, 0.004) | 0.106 | 0.002 (0.000, 0.003) | 0.121 | 0.001 (-0.001, 0.003) | 0.187 |
| **Females** | 0.003 (0.001, 0.005) | 0.001 | 0.003 (0.001, 0.004) | 0.001 | 0.003 (0.001, 0.004) | 0.003 |

Abbreviations: eGDR, Estimated glucose disposal rate; β, Regression coefficient; CI, Confidence interval.

Model 1: Adjusted for baseline age, age^2^, and sex (not included in sex-stratified analyses).

Model 2: Further adjusted for educational level, marital status, residence, smoking status, drinking status, BMI, and BMI^2^.

Model 3: Additionally adjusted for C-reactive protein, hemoglobin, dyslipidemia, diabetes, heart disease, and stroke.

**Supplementary Table 9. Associations Between Continuous eGDR and Annual Decline in Executive Function Over 7 Years**

|  | **Model 1** | | **Model 2** |  | **Model 3** |  |
| --- | --- | --- | --- | --- | --- | --- |
|  | (β, 95% CI) | ***P*** | (β, 95% CI) | ***P*** | (β, 95% CI) | ***P*** |
| **Total population** | 0.002 (0.001, 0.003) | <0.001 | 0.002 (0.001, 0.003) | <0.001 | 0.002 (0.001, 0.003) | 0.001 |
| **Males** | 0.002 (0.000, 0.004) | 0.019 | 0.002 (0.000, 0.004) | 0.022 | 0.002 (0.000, 0.004) | 0.032 |
| **Females** | 0.002 (0.001, 0.004) | <0.001 | 0.002 (0.000, 0.004) | 0.010 | 0.002 (0.000, 0.004) | 0.011 |

Abbreviations: eGDR, Estimated glucose disposal rate; β, Regression coefficient; CI, Confidence interval.

Model 1: Adjusted for baseline age, age^2^, and sex (not included in sex-stratified analyses).

Model 2: Further adjusted for educational level, marital status, residence, smoking status, drinking status, BMI, and BMI^2^.

Model 3: Additionally adjusted for C-reactive protein, hemoglobin, dyslipidemia, diabetes, heart disease, and stroke.

**Supplementary Table 10. Associations Between Continuous eGDR and Annual Decline in Episodic Memory Over 7 Years**

|  | **Model 1** | | **Model 2** |  | **Model 3** |  |
| --- | --- | --- | --- | --- | --- | --- |
|  | (β, 95% CI) | ***P*** | (β, 95% CI) | ***P*** | (β, 95% CI) | ***P*** |
| **Total population** | 0.002 (0.000, 0.003) | 0.058 | 0.002 (0.000, 0.003) | 0.080 | 0.001 (0.000, 0.003) | 0.138 |
| **Males** | 0.000 (-0.002, 0.003) | 0.952 | 0.000 (-0.003, 0.003) | 0.989 | 0.000 (-0.003, 0.002) | 0.839 |
| **Females** | 0.003 (0.000, 0.005) | 0.019 | 0.003 (0.000, 0.005) | 0.026 | 0.002 (0.000, 0.005) | 0.039 |

Abbreviations: eGDR, Estimated glucose disposal rate; β, Regression coefficient; CI, Confidence interval.

Model 1: Adjusted for baseline age, age^2^, and sex (not included in sex-stratified analyses).

Model 2: Further adjusted for educational level, marital status, residence, smoking status, drinking status, BMI, and BMI^2^.

Model 3: Additionally adjusted for C-reactive protein, hemoglobin, dyslipidemia, diabetes, heart disease, and stroke.

**Supplementary Table 11. Associations Between Covariates and Cognitive Decline in the Total Population**

| **Term** | **β(95%CI)** | ***P*-value** | **Standardized β(95% CI)** | **ΔR^2^** |
| --- | --- | --- | --- | --- |
| **eGDR*time** | 0.002 (0.001, 0.003) | 0.002 | 0.011 (0.004, 0.018) | 0.019 |
| **eGDR** | 0.017 (-0.045, 0.080) | 0.586 | 0.029 (0.009, 0.049) | 0.002 |
| **eGDR^2^** | -0.001 (-0.004, 0.003) | 0.772 | -0.002 (-0.018, 0.013) | 0.001 |
| **Time** | -0.080 (-0.093, -0.068) | <0.001 | -0.145 (-0.151, -0.138) | NA |
| **Age** | 0.052 (0.032, 0.073) | <0.001 | 0.427 (0.262, 0.592) | 0.001 |
| **Age^2^** | -0.690 (-0.873, -0.506) | <0.001 | -0.615 (-0.779, -0.451) | 0.002 |
| **Female** | -0.392 (-0.439, -0.344) | <0.001 | -0.180 (-0.202, -0.158) | 0.016 |
| **BMI** | 0.083 (0.061, 0.105) | <0.001 | 0.302 (0.222, 0.381) | 0.005 |
| **BMI^2^** | -0.238 (-0.321, -0.156) | <0.001 | -0.221 (-0.298, -0.145) | 0.003 |
| **Residence** | 0.291 (0.256, 0.325) | <0.001 | 0.127 (0.112, 0.142) | 0.017 |
| **Educational level** |  |  |  | 0.089 |
| **Elementary school or below** | -1.031 (-1.176, -0.886) | <0.001 | -0.953 (-1.088, -0.819) | NA |
| **Secondary school** | -0.300 (-0.446, -0.155) | <0.001 | -0.278 (-0.412, -0.143) | NA |
| **Marital_status Others** | -0.119 (-0.171, -0.066) | <0.001 | -0.110 (-0.158, -0.061) | 0.003 |
| **Smoking** | 0.013 (-0.033, 0.058) | 0.576 | 0.006 (-0.015, 0.026) | 0.002 |
| **Drinking** | -0.003 (-0.044, 0.038) | 0.897 | -0.001 (-0.018, 0.015) | 0.001 |
| **Dyslipidemia** | 0.035 (0.001, 0.068) | 0.043 | 0.016 (0.001, 0.031) | 0.002 |
| **Diabetes** | -0.044 (-0.090, 0.002) | 0.060 | -0.015 (-0.030, 0.001) | 0.002 |
| **Heart disease** | 0.075 (0.024, 0.125) | 0.004 | 0.022 (0.007, 0.037) | 0.001 |
| **Stroke** | -0.059 (-0.177, 0.058) | 0.322 | -0.007 (-0.022, 0.007) | 0.001 |
| **CRP** | -0.001 (-0.004, 0.001) | 0.187 | -0.010 (-0.024, 0.005) | 0.002 |
| **hemoglobin** | 0.002 (-0.006, 0.009) | 0.677 | 0.003 (-0.013, 0.019) | 0.001 |

Abbreviations: eGDR, Estimated glucose disposal rate; β, Beta coefficient; CI, Confidence interval; ΔR², Change in R-squared; BMI, Body Mass Index; CRP, C-reactive protein.

**Supplementary Table 12. Sex Differences in Educational Attainment Among the Study Population**

|  | Overall | Female | Male | p |
| --- | --- | --- | --- | --- |
| n | 8734 | 4678 | 4056 |  |
| Educational_level (%) | |  |  | <0.001 |
| Elementary school or below | 6126 (70.1) | 3660 (78.2) | 2466 (60.8) | |
| Secondary school | 2494 (28.6) | 983 (21.0) | 1511 (37.3) | |
| College and above | 113 (1.3) | 35 (0.7) | 78 (1.9) | College and above |
